# An investigation of the ‘female camouflage effect’ in autism using a computerized ADOS-2 and a test of sex/gender differences

**DOI:** 10.1186/s13229-016-0073-0

**Published:** 2016-01-21

**Authors:** Agnieszka Rynkiewicz, Björn Schuller, Erik Marchi, Stefano Piana, Antonio Camurri, Amandine Lassalle, Simon Baron-Cohen

**Affiliations:** Department of Psychiatry, Medical University of Gdansk, Gdańsk, Poland; Centrum Diagnozy, Terapii i Edukacji SPECTRUM ASC-MED, Gdańsk, Poland; Machine Intelligence and Signal Processing Group, Technische Universität München, Munich, Germany; Department of Computing, Imperial College London, London, UK; Chair of Complex and Intelligent Systems, University of Passau, Passau, Germany; Casa Paganini-InfoMus Research Centre DIBRIS, University of Genoa, Genoa, Italy; Department of Psychiatry, Autism Research Centre, Cambridge University, Cambridge, UK

**Keywords:** Females with autism, ADOS-2, Diagnosis, Computer application

## Abstract

**Background:**

Autism spectrum conditions (autism) are diagnosed more frequently in boys than in girls. Females with autism may have been under-identified due to not only a male-biased understanding of autism but also females’ camouflaging. The study describes a new technique that allows automated coding of non-verbal mode of communication (gestures) and offers the possibility of objective, evaluation of gestures, independent of human judgment. The EyesWeb software platform and the Kinect sensor during two demonstration activities of ADOS-2 (Autism Diagnostic Observation Schedule, Second Edition) were used.

**Methods:**

The study group consisted of 33 high-functioning Polish girls and boys with formal diagnosis of autism or Asperger syndrome aged 5–10, with fluent speech, IQ average and above and their parents (girls with autism, *n* = 16; boys with autism, *n* = 17). All children were assessed during two demonstration activities of Module 3 of ADOS-2, administered in Polish, and coded using Polish codes. Children were also assessed with Polish versions of the Eyes and Faces Tests. Parents provided information on the author-reviewed Polish research translation of SCQ (Social Communication Questionnaire, Current and Lifetime) and Polish version of AQ Child (Autism Spectrum Quotient, Child).

**Results:**

Girls with autism tended to use gestures more vividly as compared to boys with autism during two demonstration activities of ADOS-2. Girls with autism made significantly more mistakes than boys with autism on the Faces Test. All children with autism had high scores in AQ Child, which confirmed the presence of autistic traits in this group. The current communication skills of boys with autism reported by parents in SCQ were significantly better than those of girls with autism. However, both girls with autism and boys with autism improved in the social and communication abilities over the lifetime. The number of stereotypic behaviours in boys significantly decreased over life whereas it remained at a comparable level in girls with autism.

**Conclusions:**

High-functioning females with autism might present better on non-verbal (gestures) mode of communication than boys with autism. It may camouflage other diagnostic features. It poses risk of under-diagnosis or not receiving the appropriate diagnosis for this population. Further research is required to examine this phenomenon so appropriate gender revisions to the diagnostic assessments might be implemented.

## Background

Autism spectrum condition (autism) affects 1 % of the population [1–3]. It is diagnosed more frequently in boys than in girls, with the 4:1 ratio widely cited [4–7]. However, the studies of the last two decades show a trend of decreasing male predominance. The recent studies show that this proportion may be lower: 2.0–2.6:1 [8–10] which can be attributed to better knowledge and improved diagnostic skills of mental health professionals as well as revised diagnostic tools and criteria. Girls are diagnosed with autism less frequently than their male counterparts, which also results in less research conducted in this patient group to address the issue of sex/gender-related differences in functioning or clinical manifestation [10–13].

Autism is a complex neurobiological condition, the aetiology of which has not been fully determined. The research so far has pointed out to the numerous genetic, neuroanatomical and immune abnormalities as well as neurotransmitter dysfunction [14–17]. One current, interesting theory assumes that the level of oxytocin, being a neuromodulator involved in social bonding, exerts a protective effect on manifestation of autistic traits in girls [18]. A second related possibility concerns the hormone fetal testosterone (FT), which is produced in higher quantities in typical males than typical females, and is elevated in children with autism [19]. Lower FT may thus also exert a protective effect on the manifestation of autistic traits in girls. To a certain degree, these theories can explain why autistic traits are fewer in many high-functioning girls with autism as compared to boys with autism [20, 21]. As a result, girls may not be appropriately diagnosed or be diagnosed with autism at a later age, which has been also reported recently in the research on Polish high-functioning population of autism adolescents [22].

The deficits typical of autism are manifested in the following areas: communication, social competence and rigid, stereotypic behaviours [23]. The symptoms may be noticed and diagnosed even in early childhood [24]. However, they may also become manifested in later age, when the social demand and requirements exceed the limited functional capabilities of an autism individual. A recent study of high-functioning Polish females with autism in adolescence (the term ‘high functioning’ refers to an individual with autism who is verbally fluent, with average or above average intelligence) shows that they present a more atypical sensory profile than males with autism [22]. Thus, including sensory profile in the new Diagnostic and Statistical Manual of Mental Disorders, Fifth Edition (DSM-5) diagnostic criteria [25] can have positive implications on autism diagnosis in females. Females with autism have greater self-awareness and make greater effort to camouflage their deficits [22, 26, 27]. Diagnostic and screening tests in autism such as ADOS-2 (Autism Diagnostic Observation Schedule Second Edition [28] which has been referred to as the “gold standard” observational assessment for diagnosing autism), AQ (Autism Spectrum Quotient) [29] and SCQ (Social Communication Questionnaire) [30] are developed based on the phenotype typical of boys with autism, which does not include many characteristics of girls with autism [20, 22]. Despite the SCQ specifically targeting the social and communication domains, it has not yet been assessed for possible gender differences in its scores. The tests in autism may be biased toward identifying autism predominantly in males [31]. For instance, males with autism exhibit more repetitive behaviours than females with autism [32]. Thus, if repetitive behaviour is used as critical diagnostic criteria, females with autism might be missed. Although Van Wijngaarden-Cremers et al. [32] did not observe differences between boys and girls with autism in the social and communicative domains, Rynkiewicz and Łucka [22] recently found gender differences on the ADOS-2 scores related to those domains in a Polish sample. Thus, it remains unclear whether males and females with autism differ only in the repetitive domain or also on the social behaviour and communication dimension. The exploration of the non-verbal communication (gestures) in autism and comparison between sex/gender is important for two reasons. First, children with autism show deficits in the development of gestural communication [33]; they gesture less and at lower rates compared to both typically developing children and also other children who are developmentally delayed [34]. Second, gestures are coded under the communication section of ADOS-2 algorithm. The better an individual performs on gesturing, the lower the autism score he or she receives under this item on the algorithm. The recent gender differences on the scores in ADOS-2 under this non-verbal communication domain in a Polish sample of high functioning adolescents with autism [22] require further investigation of this dimension.

Since Polish diagnostic and screening measures tap the symptomatology associated with autism, and since the intensity of autistic traits and the emotional competence of Polish children with autism are not widely described in the international literature, we also present the results obtained on these measures with particular focus on parent-report screening measurements as they include the questions on the communication of a child, both verbal and non-verbal (gestures) as well.

The primary aim of this study is to present an innovative computerized technique to objectively evaluate the non-verbal modality of communication (gestures) during two demonstration tasks of ADOS-2 (Autism Diagnostic Observation Schedule, Second Edition). The authors describe a new technique allowing automated coding of non-verbal mode of communication (gestures) and offering the possibility of objective, evaluation of gestures, independently of human judgment and automatically measured participants’ gestures, allowing computation of a “Gesture Index” (GI). This GI was compared between males and females with autism. The authors tested if females with autism had a higher GI compared to males with autism. To our best knowledge, no such studies have been carried out so far. This gap in the literature is filled in with the study described below.

### Participants

The study group consisted of 33 high-functioning Polish girls and boys with formal diagnosis of autism or Asperger syndrome who were recruited into the autism spectrum conditions (ASC)-Inclusion project [23]. The project’s inclusion criteria in Poland were as follows: age range of 5–10 years; intelligence quotient (IQ) average or above; verbally fluent native speakers of Polish given the study being executed in Poland; formal diagnosis of autism or Asperger’s syndrome based on International Statistical Classification of Diseases and Related Health Problems, Tenth Edition (ICD-10) [35] or Diagnostic and Statistical Manual of Mental Disorders, Fourth Edition (DSM-IV) [36], the criteria made by a psychiatrist and a clinical psychologist and children with no co-occurring serious mental, neurological or paediatric illnesses (e.g., epilepsy). For the purpose of this study and to meet the inclusion criteria, it was sufficient that the IQ of a child was previously tested; this fact was documented in the medical records and the IQ results were average or above, provided either as a numerical score or as a written form which is a standard in Polish medical records. Polish researchers had access to a child’s medical records. The relevant clinical information is included in Table 1. The enrolled participants were children from Child and Adolescent Mental Health Services and Autism Clinics in Pomeranian, Mazovian and Subcarpathian voivodeships (Poland). As outlined, parents of our participants were also a part of the study. They participated in the assessments regarding their children which are referred to in the subsequent part of the paper.

**Table 1:** Demographic table including the age, IQ scores and ADOS-2 scores

	Age		IQ score		ADOS-2 score	
	Females	Males	Females	Males	Females	Males
*N*	16	17	13	16	16	17
Mean	8.06	8.23	109.58	112.31	8.94	9.71
SD	1.57	2.05	11.70	13.10	2.46	2.95

*N* number of included participants in the sample, *SD* standard deviation

## Methods

The research was approved by Independent Bioethical Committee by Medical University of Gdańsk and local Internal Review Board at the District Chamber of Physicians in Gdańsk, both in Poland (no. KB-20/13) and the European Commission Ethics Review Team (ASC-Inclusion project, grant agreement no. 289021), under FP7-ICT for smart and personalized inclusion. The authors of all diagnostic tools used in the studies gave their permission to such use and appropriate licenses were issued to the author-reviewed Polish research translation of the ADOS-2 and SCQ tests by the publisher, WPS (Western Psychological Services, USA). Informed written consent was obtained for all participants in accordance with procedures approved by the above Research Ethics Committees.

### Measures

**Fig. 1:**
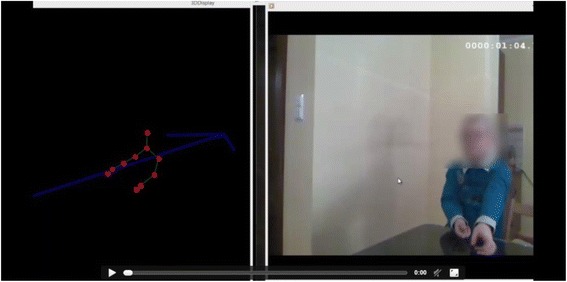
Two demonstration tasks of ADOS-2 sessions recorded with Kinect

In this study, we focused on two demonstration activities of ADOS-2 [28] to collect automated, computerized data on gestures, independent of human judgment. This is a semi-structured, standardized assessment of communication, social interaction, play/imaginative use of materials and restricted and repetitive behaviours. Thirty-three high-functioning children with autism were tested during two demonstration activities of Module 3 of the author-reviewed Polish research translation ADOS-2. The ADOS-2 was administered in Polish and coded using Polish codes. Automated measurement of gestures was performed during the Demonstration Task and the Cartoons Task (Fig. 1): the Demonstration Task assesses the participant’s ability to communicate about a familiar series of actions using gestures or mime with accompanying language. The Cartoons Task provide an opportunity to observe the way in which a participant narrates a story, uses gestures to enact events and integrates gestures with gaze and language. The automated measurement is performed by a software application designed and build on the EyesWeb software platform (http://www.infomus.org/eyesweb_eng.php) [37], by the Casa Paganini—InfoMus Research Centre of University of Genoa, partner in ASC-Inclusion [23]. EyesWeb software platform supports the design and development of real-time multimodal systems and applications, integrating a wide number of (fully synchronized) input devices including motion capture systems, various types of professional and low cost video cameras, game interfaces (e.g. Kinect, Wii), multichannel audio input (e.g. microphones), and analog inputs (e.g. for physiological signals). EyesWeb outputs include multichannel audio, video, analog devices and robotic platforms. Particularly useful features of EyesWeb include the support of real-time synchronized recordings of multimodal channels and the software libraries for non-verbal expressive gesture analysis and non-verbal social signals analysis. In this specific application, we used the Microsoft Kinect sensor for the automated gesture analysis task.

The Kinect allows one to interact with the computer while using body movement. It was connected to an ASUS G750 computer with Windows 8 OS, using the EyesWeb platform. The Kinect and the video camera were placed in front of the table where an ADOS-2 session was administered to each child. Every tested child was facing the Kinect. Three researchers were present in a testing room: researcher #1 who was administrating ADOS-2 to a child, researcher #2 who was recording two demonstration activities with the Kinect connected to the computer application and researcher #3 who was recording the whole ADOS-2 session with a video camera. Researcher #2 started and ended recordings with the Kinect and the computer application on researcher #1’s very precise signal. It marked the start and the end of each demonstration activity. All the recordings were double checked by the researchers after each session to make sure that the start and the end of the demonstration activities were recorded correctly for further analysis. In the output, the application we used returns with coordinates in the *X*, *Y*, and *Z* dimensions of ten characteristic points of a child being recorded (head, neck, left shoulder, right shoulder, left elbow, right elbow, left wrist, right wrist, left palm with the fingers and right palm with the fingers). Each point is shown in Fig. 1. The numerical data from the recordings of each point was calculated. The scores of each individual point representing individual part of the child’s body were computed from the precisely marked start to the end of each demonstration activity. The results of all 10 points, for each demonstration activity individually and as a sum of two activities together were presented for each gender separately and also compared between two genders. If any of the coordinates were missing or incomplete on the Kinect recordings, we did not take this data to the final analysis and the comparison.

As a result, we compared 10 girls with autism and 16 boys with autism on gestures (Tables 2, 3 and 4). We did not differentiate gestures on descriptive, conventional, instrumental, informational, emotional or emphatic (the last ones are integrated into an individual’s speech). The computer application summed all the gestures together during two ADOS-2 activities. During a typical, standard ADOS-2 assessment—not computerized—all noted gestures are also summed up, and then, a code is provided to the final algorithm. Although in a standard way an examiner (a human) differentiates the gestures on different types, the final code to the ADOS-2 algorithm represents a sum of all the types of gestures observed and noted during a session. It would be a very interesting next step in future research of the automated coding to distinguish different types of gestures in ADOS-2. Such a sophisticated automated coding may provide more detailed information on each different type of gestures presented during an ADOS-2 session.

**Table 2:** The length of gestures [mm] in autism group according to sex/gender (10 females, 16 males)

	Demonstration Task		Cartoons		Sum of two activities	
	Girls	Boys	Girls	Boys	Girls	Boys
Mean	191,419.9	192,023.8	45,607.41	43,459.44	237,027.26	235,483.28
SE	±30,769.97	±29,795.39	±32,788.59	±24,804.43	±59,295.35	±40,124.21
Mann-Whitney *U*	74.00		71.00		78.00	
*p*-value	0.78		0.66		0.94

**Table 3:** The time of gestures [s] in autism group according to sex/gender (10 females, 16 males)

	Demonstration Task		Cartoons		Sum of two activities	
	Girls	Boys	Girls	Boys	Girls	Boys
Mean	35.6	47.6	37.8	45.3	72.9	92.9
SE	±4.35	±8.77	±4.85	±5.27	±7.98	±10.64
Mann-Whitney *U*	70.00		65.00		60.00	
*p*-value	0.62		0.42		0.31

**Table 4:** The Gesture Index, length to time [mm/s], according to sex/gender in autism group (10 females, 16 males)

	Demonstration Task		Cartoons		Sum of two activities	
	Girls	Boys	Girls	Boys	Girls	Boys
Mean	5,485.3	4,521.8	965.0	1,318.2	3,092.72	2,806.73
SE	±524.70	±585.12	±606.51	±857.14	±547.53	±606.46
Mann-Whitney *U*	56.00		70.00		66.00	
*p*-value	0.22		0.62		0.48	

In this study, the “time of gestures” refers to the duration of all gestures that were automatically measured and then summed up for each separate activity (either during the Demonstration activity or the Cartoons activity). It represents the mean duration of gestures (in seconds). The “length of gestures” is the sum of the lengths of all the trajectories (in mm) in a given gesture, and finally, the “Gesture Index” is the average speed of the joints over a gesture during both demonstration activities. There is a rationale behind our selected measurements. The gesture length is related to the way a person occupies the surrounding space with her body (see Laban’s personal space [38, 39]) and related to emotion expression and recognition: for example, fear may be characterized by defensive postures (i.e. keeping hands near the trunk or the head) and shorter length movements, whereas anger may imply wider and faster (short in time, impulsive) movements [40]. The duration (in time) of a gesture is used to calculate the Gesture Index which is related to the energy spent by the subject during a gesture. This quantity is important in emotion recognition, as stated by Wallbott and Bodily [41] that showed how movement activity is a relevant feature in recognizing emotion from full-body movement.

In order to determine the children’s functioning level and their emotion recognition competence, the Polish versions of the following tests were used: AQ Child [42], the Faces Test [43] and the Eyes Test (Child) [44]. The AQ Child consists of 50 statements related to different aspects of the child’s daily functioning. It is used to assess the intensity of autistic traits in an individual. The Eyes Test (Child) consists of 37 black-and-white photographs of human eyes (both male and female) showing different emotions. Four emotions are itemized under each photo, and only one of them is correct. The parents of the children also completed the Polish version of SCQ [30]. This is a 40-item parent-report screening measure that taps the symptomatology associated with autism. The items are administered in a *yes/no* response format. There are two forms: SCQ Lifetime which refers to the individual’s entire developmental history and SCQ Current which refers to the individual’s behaviours during the most recent 3-month period and the parents who were participating in the study completed both SCQ forms. The SCQ analysed as a whole, assesses the intensity of autistic traits. Since the SCQ subscores in conjunction with the total score may prove useful in the evaluation of group differences in research usage according to the SCQ Manual [30], we investigated the subscores and domains as well: reciprocal social interaction, communication and restricted, repetitive and stereotypic pattern of behaviour.

### Statistical analysis

Statistical analysis included mean values or mean frequencies of analysed parameters in order to determine differences that can be directly attributed to sex. The statistical comparisons were made using the Mann-Whitney *U* test. All statistical analyses were carried out using SPSS software (IBM SPSS Statistics v. 21).

## Results and discussion

We focused on the three key aspects while the gestures were recorded during two demonstration activities of ADOS-2 sessions: motor activity when demonstrating the toothbrushing skill under the Demonstration Task, motor activity when re-telling the story under the Cartoons and motor activity of both activities added together. In each aspect, we paid attention to the length by which the respective body parts (each point individually out of 10 points representing the body parts mentioned above) were moved in gesturing (Table 2), the time a child spent performing the task (Table 3) and the Gesture Index which is the length to time ratio of the above (Table 4). Based on the Gesture Index in our study, the results showed that girls with autism tended to use gestures more vividly than boys with autism. The term “vivid” refers to gestures that in our interpretation are longer gestures but presented in shorter time (higher Gesture Index for girls with autism than Gesture Index for boys with autism). As a result, such gestures might be noticed by an observer (a human) as the gestures with increased energy.

On the Faces Test, children answered all questions in most cases (99 % of provided answers). The autism group presented itself with significantly lower scores of girls than boys. During the Eyes Test (Child), children tended to stop working earlier, typically leaving five questions unanswered (89 % of provided answers). On average, the children named half of the presented emotions properly. The percentage of correct answers by boys with autism was significantly higher than that of the one of girls with autism. Based on parental answers to AQ Child, the scores were high and confirming the presence of autistic traits. Furthermore, there were no significant score differences between boys with autism and girls with autism on this test. The summary for participant scores by gender on the AQ Child, the SCQ, SCQ Lifetime and SCQ Current and the proportion of correct trials for the Eyes and Face Tests are presented in Table 5.

**Table 5:** Summary table for participant scores by sex/gender

	SCQ Lifetime		SCQ Current		AQ Child		Eyes Test		Faces Test	
	Males	Females	Males	Females	Males	Females	Males	Females	Males	Females
*N*	16	16	16	16	14	12	12	13	15	14
Mean	22.94	23.31	14.19	14.13	31.86	32.58	0.54	0.40	0.80	0.72
SD	6.98	5.39	3.90	5.94	7.77	8.75	0.18	0.14	0.08	0.09

*N* number of included participants in the sample, *SD* standard deviation

Based on parental answers in the SCQ, we found that the general lifetime score of autistic children shows the high intensity of autism traits, whereas the current functioning score is strongly suggestive of the autism traits, as the results are close to the cutoff point. Social deficits for both girls with autism and boys with autism occur currently over two times less frequently than over the lifetime. Similarly, communication ability has significantly improved and, currently, the deficits in this domain are less frequent. It is interesting to note that the current communication skills of boys with autism are significantly better than those for girls with autism as reported by their parents. The obtained results also confirm that the number of stereotypic behaviours in boys with autism significantly decreased over life, whereas it remains at comparable level in girls with autism.

### Limitations and future directions

The study describes a new technique that allows automated coding of non-verbal mode of communication and offers the possibility of objective, evaluation of gestures, independent of human judgment. The study has some limitations. So far, such research with computerized automated coding of gestures has not been done on the whole ADOS-2 session. It is advisable to replicate this study in the future to extend it to the whole ADOS-2 session and to include autistic children with co-occurring illnesses too. In this study, we were limited due to the project’s inclusion criteria. This future direction is supported by the other recently published study on high-functioning Polish females with autism in their adolescent years by Rynkiewicz and Łucka which shows significantly high comorbidity of anxiety and depression among females with autism but also fewer behavioural autistic features than Polish high-functioning autism male adolescents under communication section in both Autism Diagnostic Observation Schedule (ADOS) and ADOS-2 algorithms during a standard assessment [22]. The other limitation is that the Gesture Index (GI) might seem different from how gestures are typically coded and understood, particularly in the context of ADOS-2. The authors will aim in the future study to compare the GI result from the whole computerized ADOS-2 session with the sum of all the types of gestures recorded and noted by an examiner (a human) during a standard, non-computerized ADOS-2 session. Since there has been no other study published so far on both Polish research translation of SCQ Lifetime and SCQ Current, it is recommended to investigate further this parent-report screening measurement as well. It is advisable to research the presented phenomena with GI further and also across the other cultures.

## Conclusions

In this study, we found that high-functioning females with autism present better on non-verbal (gestures) mode of communication than boys with autism. This may be because they are effective at camouflaging other diagnostic features. This may pose risk of under-diagnosis or not receiving the appropriate diagnosis for this population. In this study, the girls with autism present the higher Gesture Index than boys with autism, which is interpreted as gestures with increased energy, more “vivid” and thus probably more noticeable by an examiner (a human); as a result, such non-verbal communication may be noted as not autistic. In the present study, girls with autism present difficulties on the Faces Test; however, they similarly have more “vivid”, noticeable gestures on computerized measures when compared to boys with autism while at the same time on the parent-report screening tests, there is either the absence of difference (AQ) or parents rate the boys with autism as having less affected communication (SCQ). Thus, the study also raises a question on the parent-report screening measures where parents are given the instrument and asked to answer the questions without direct supervision: Do parents take under consideration the non-verbal communication (gestures) when they judge their child’s communication skills or perhaps parent’s judgment is biased by only the verbal communication of their child? The finding of this study supports the other research and clinical reports on Polish females with autism [22, 27] that high-functioning females with autism might present greater determination over lifetime to learn social norms and nuances of communication, both verbal and non-verbal (gestures) than high-functioning males with autism. The implications of this are important because the high-functioning adolescent females with autism are at risk in older age of receiving non-spectrum classification in ADOS-2 due to good performance on communication domains while their developmental history and clinical manifestation confirm the autism spectrum [22]. Further research is required across the cultures to examine this phenomenon.

These results are part of a larger research programme of the authors on high-functioning females with autism. The results might be valuable for future revisions of diagnostic assessments. The authors hope that this information may contribute to the increased diagnosis of autism spectrum conditions in girls, which can ultimately improve the diagnostic process and efficacy of interventions in this group.

## Abbreviations

ADOS: Autism Diagnostic Observation Schedule
ADOS-2: Autism Diagnostic Observation Schedule, Second Edition
AQ Child: Autism Spectrum Quotient (Child)
ASC: autism spectrum conditions
DSM-5: Diagnostic and Statistical Manual of Mental Disorders, Fifth Edition
DSM-IV: Diagnostic and Statistical Manual of Mental Disorders, Fourth Edition
ICD-10: International Statistical Classification of Diseases and Related Health Problems, Tenth Edition
IQ: intelligence quotient
SCQ: Social Communication Questionnaire
